# Human Exposure to Chlorinated Organophosphate Ester Flame Retardants and Plasticizers in an Industrial Area of Shenzhen, China

**DOI:** 10.3390/ijerph19053126

**Published:** 2022-03-07

**Authors:** Yunlang Liu, Tingting Zhu, Zuoming Xie, Chen Deng, Xiujuan Qi, Rong Hu, Jinglin Wang, Jianyi Chen

**Affiliations:** 1 Hubei Key Laboratory of Yangtze Catchment Environmental Aquatic Science, School of Environmental Studies, China University of Geosciences, Wuhan 430074, China; yunlangliu@foxmail.com; 2 State Environmental Protection Key Laboratory of Managing Technology of Drinking Water Source, Shenzhen Key Laboratory of Emerging Contaminants Detection & Control in Water Environment, Shenzhen Academy of Environmental Science, Shenzhen 518001, China; dengchen@meeb.sz.gov.cn (C.D.); qixiujuan@meeb.sz.gov.cn (X.Q.); hurong@meeb.sz.gov.cn (R.H.); wangjinling@meeb.sz.gov.cn (J.W.); chenjianyi@meeb.sz.gov.cn (J.C.); 3 State Key Laboratory of Biogeology and Environmental Geology, China University of Geosciences, Wuhan 430074, China

**Keywords:** organophosphate esters, industrial area, internal exposure, heath risk

## Abstract

Human exposure to organophosphate esters (OPEs) is more pervasive in industrial areas manufacturing OPE-related products. OPE exposure is of great concern due to its associations with adverse health effects, while studies on OPE exposure in industrial districts are scarce. This study aimed to assess human exposure to OPEs in a typical industrial area producing large amounts of OPE-related products in Shenzhen, China. Tris (2-chloroethyl)-phosphate (TCEP), tris (2-chloroisopropyl) phosphate (TCPP) and other common OPEs were analyzed in urine (*n* = 30) and plasma (*n* = 21) samples. Moreover, we measured five OPE metabolites (mOPEs) in plasma samples (*n* = 21). The results show that TCPP and TCEP are dominant compounds, with moderate to high levels compared with those reported in urine and plasma samples from other regions. In addition, di-n-butyl phosphate (DnBP) and diethyl phosphite (DEP) were frequently detected in plasma samples and could be considered as biomarkers. Risk assessment revealed a moderate to high potential health risk from TCEP exposure. Our results provide basic data for human exposure to OPEs in industrial areas and call for the prevention and mitigation of industrial chlorinated OPE pollution.

## 1. Introduction

Recently, some widely used brominated flame retardants, e.g., polybrominated diphenyl ethers (PBDEs), were banned or restricted due to their persistence, long-range atmospheric transport, bioaccumulation and potential adverse effects on mammals [1]. As alternatives to PBDEs, the consumption of organophosphate esters (OPEs) has surged, with an annual increase of 15% in China [2]. OPEs are applied as flame retardants, plasticizers and defoamers in products. Because they are mainly added to materials by doping and mixing rather than by chemical bonding, OPEs could easily enter the environment. Consequently, OPEs have been widely detected in various environmental media globally [3], including sediments from the Futian Mangrove Nature Reserve [4], indoor dust [5], human urine samples from primary school students and primiparas [6,7] and human blood samples from Shenzhen [8]. These findings revealed ubiquitous environmental occurrences and human exposure to OPEs. However, information regarding human exposure to OPEs in industrial areas is rather scarce.

Epidemiological studies have reported OPE exposure potentially associated with health effects, such as altered hormone levels [3], metabolic syndrome [9], sphingolipid homeostasis [8], oxidative stress [10,11], neurodevelopment [12] and even cancers [13]. Given the increasing industrial demand and potential health effects of OPEs, human exposure to OPEs is of great concern, especially for high-risk populations such as residents in industrial areas.

The Pearl River Delta (PRD) in southern China, at which lie industrial clusters of electronics, building materials, textiles, coatings and other plastic products, is the main OPE-applying and high-OPE exposure-risk area in China. Previous studies reported the worldwide highest concentrations of OPEs among the rivers and fish in the PRD [2]. Shenzhen, located on the south coast of the PRD, is a rapidly urbanized and highly industrialized city. Compared with other cities of the PRD, Shenzhen possesses more agglomerated industries, which manufacture large amounts of OPEs or OPE-related products. Approximately 70% of the factories in Shenzhen are distributed in the Maozhou River Basin of Bao’an District.

Therefore, we hypothesized that residents from the industrial area in Shenzhen might be at a higher exposure risk than the general population. The present study aimed to (1) determine commonly used OPEs and their metabolites in urine and plasma samples of residents from an industrial area and (2) calculate the estimated daily intakes and assess potential health risks.

## 2. Materials and Methods

### 2.1. Sample Collection

**Figure A1:**
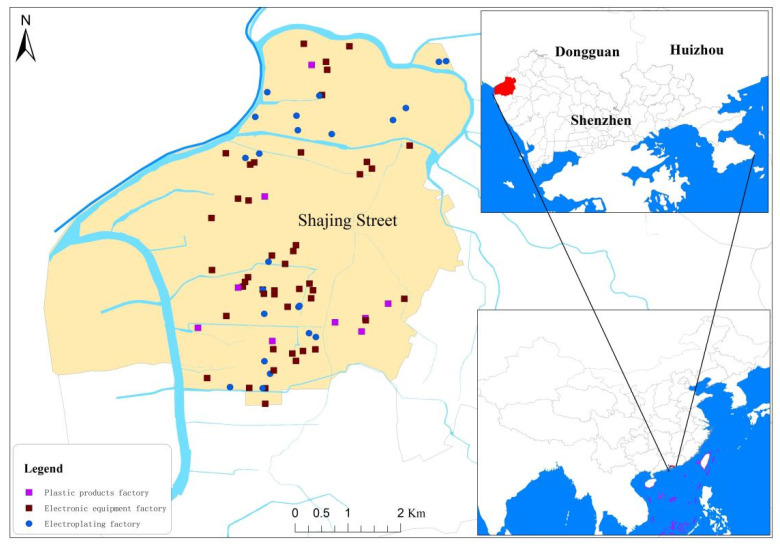
Sampling area map.

During October 2020, 30 urine samples and 21 plasma samples of residents from Shajing Street in the Maozhou River Basin (Figure A1) were collected for OPE analysis. We collected voluntary informed consent of the participants through the Shenzhen Bao’an District People’s Hospital when they had a routine physical examination. Urine samples (20 mL) and blood samples (10 mL) were collected in glass bottles. The samples were transported in a box with dry ice to the laboratory and stored at −20 °C for further analysis. The volunteer’s information is shown in Table A1. The ethical committee of the School of Public Health (Shenzhen), Sun Yat-sen University, approved the present study.

### 2.2. Standards and Reagents

A full list of the 17 target OPE compounds and 5 mOPE compounds, along with their full chemical names, physicochemical properties and respective manufacturers, can be found in Table A2. Internal standards, including TiBP_-d27_, TBOEP_-d27_ and TMP_-C13_, were purchased from Toronto Research Chemicals Inc. (Toronto, ON, Canada). The purities of all analytical standards used in this study were ≥95%.

Methanol and dichloromethane of HPLC grade were purchased from Merck, Germany. Ultrapure water was obtained from a Millipore Waters Milli-Q water purification system. An Agilent 1260-6460 high-performance liquid chromatography-triple quadrupole mass spectrometry instrument (HPLC-MS/MS) was obtained from Agilent Technologies Co., Ltd. (Shanghai, China). An HZQ-Q constant temperature oscillator was obtained from Donglian Electron Technology Exploiter Co., Ltd. (Harbin, China). A vacuum freeze drier was obtained from Pharmaceuical Machinery Co., Ltd. (Shanghai, China). A WAX Plus column (60 mg, 3 mL) was purchased from Waters Corp. (Milford, MA, USA). Other materials included automatic Multi-channel Solid Phase Extraction (Thermo Fisher, Waltham, MA, USA), a 0.45 μm glass fiber filter membrane (GF/F Whatman), organomationn-evap 24 Tube Water Bath Nitrogen Blower (Organomation, Berlin, MA, USA) and a high-speed bench centrifuge (Sigma Aldrich, Shanghai, China).

### 2.3. Sample Pretreatment and Instrumental Analysis

OPEs in the urine and plasma samples were extracted according to published methods [12,14] with minor modifications.

Briefly, for urine samples, 1 mL urine was added with standard substitute to obtain a concentration of 100 ng/mL and for purification with solid-phase extraction. First, 2 mL of methanol solution containing 5% ammonia was added to the column, and the solution dripped naturally. When the solution was equal to the substance in the column, 2 mL of ultrapure water containing 8‰ formic acid was added for the same operation. Second, 1 mL of spiked urine was passed through the activated small column, and the effluent was discarded. Third, we washed the column with 2 mL of ultrapure water containing 8‰ formic acid and discarded the leaching solution. Subsequently, after elution, a vacuum pump was used to pump for about 15 min to ensure the drying of the solid-phase extraction column. After drying, the solid phase extraction column was eluted with 1 mL of methanol solution containing 5% ammonia and 1 mL of acetonitrile with 2 min. Lastly, after the solid-phase extraction of the sample, the eluent was dried under mild nitrogen flow, concentrated to 1 mL with ultrapure water, and was stored at −20 °C for mass spectrometry analysis.

For plasma samples, 800 μL acetonitrile (containing 1% formic acid), 200 μL plasma and 100 ng/mL standard substitutes were added to a 1 mL PRiME HLB column. Then, the standard substitute was fully mixed in the column and allowed to drop naturally under the action of gravity. After the filtrate was blown and concentrated with mild nitrogen flow, the volume was fixed to 0.2 mL with ultrapure water and stored at −20 °C for mass spectrometry analysis.

The concentrations of OPEs were detected via an Agilent 1260 HPLC coupled to an Agilent 6460 MS/MS with positive electrospray ionization (ESI+). For chromatographic conditions, separation of analytes was conducted using an Agilent ZORABX SB-C18 (6 mm × 150 mm × 5 μm) column. The column temperature was 35 °C. Mobile phase A was methanol, and mobile phase B was ultrapure water containing 5 mM ammonium acetate. The flow rate, injection volume and ion source temperature were set at 250 µL/min, 10 µL and 550 °C, respectively. The gradient elution procedure is shown in Table A3. For mass spectrometry, we used an electrospray ionization source (ESI) with the ion source temperature at 550 °C, and the ionization mode was positive ionization mode. The capillary voltage was 3500 V, and the atomizer pressure was 45 psi. The carrier gas was high-purity nitrogen, the carrier temperature was 330 °C, the flow rate was 9.5 L/min and multiple reaction monitoring mode (MRM) detection was performed. A standard solution of an analyte with a concentration of 500 μg/L was prepared, the parent and daughter ions of each compound were determined by the total ion flow diagram and the corresponding mass spectrometry results and the voltage (Fragment) and collision energy (CE) were optimized according to the response. The optimized qualitative/quantitative ion mass spectrometry parameters are shown in Table A4.

### 2.4. Quality Assurance and Quality Control

The analytical method was validated based on precision, a spike–recovery experiment, blank contamination, the linearity of the calibration curve, method detection limits (MDLs) and method quantitation limits (MQLs). A standard addition method based on six concentration levels (5, 10, 50, 100, 500 and 1000 ng/mL) was used for individual target compounds, and recoveries of OPEs in urine and plasma samples were 58–126% and 64–131%, respectively (Table A5).

Signal-to-noise ratios of 3 and 10 were assumed to correspond to the MDLs and MQLs, respectively. Both were based on the lowest concentration and signal-to-noise ratio in actual samples. In urine samples, MDLs and MQLs ranged from 0.02 to 0.3 ng/mL and 0.08 to 1 ng/mL for all compounds, respectively. In plasma samples, MDLs and MQLs ranged from 0.08 to 1 ng/mL and 0.4 to 3 ng/mL (Table A5). Moreover, in order to reduce the potential background interference, the glassware used in this study (such as a brown bottle and flask) were immersed in chromic acid for more than 2 h, then washed with ultrapure water (>18.2 MΩ·cm) and roasted at 450 °C for 4.5 h, as far as possible to reduce the pretreatment process of experimental pollution.

### 2.5. Heath Risk Assessment

Based on methods in the published literature [13,15], we calculated the estimated daily intakes (*EDI*) and compared them with the tolerable intake value (*R*f*D*) to assess potential health risks (*HQ*). An *HQ* greater than 1 indicates that the contaminant poses a potential non-carcinogenic risk to human health; the formula is as follows:

(1) $$EDI=\frac{UC\times UV}{f\times bw}$$

(2) $$HQ=\frac{EDI}{RfD}$$

where *EDI* (μg·kg·*bw*^−1^·day^−1^) is the estimated daily intake of OPEs; *UC* (μg·L^−1^) is the concentration of various OPEs in individual urine; *UV* (L·day^−1^) is the daily excretion of human urine; *bw* (kg) is the body weight; *f* is the mole fraction of OPEs absorbed and excreted in the human body; RfD (ng·kg·*bw*^−1^·day^−1^) is the tolerable daily intake. The values of *UV* (2 L·day^−1^), *bw* (55 kg), *f* (0.18) and *R*f*D* (TCPP, 5000 ng·kg·*bw*^−1^·day^−1^, TCEP 2200 ng·kg·*bw*^−1^·day^−1^, TBOEP 1500 ng·kg·*bw*^−1^·day^−1^) were adopted from a previous study [15].

### 2.6. Data Analyses

All statistical analyses were performed using SPSS version 18.0 software (SPSS Inc., Chicago, IL, USA). Pictures and other illustrations were developed using Origin 19 (OriginLab Corp., Northampton, MA, USA). In the process of concentration data analysis, the value below MQLs was set to 1/2 of the MQL, and the value below MDLs was regarded as zero.

## 3. Results and Discussion

### 3.1. Concentrations and Profiles of OPEs in Urine and Plasma Sample

The detection frequencies (DFs) and concentrations of detectable OPEs measured in urine and plasma samples are presented in Table 1. In urine samples, four detectable OPEs including TCPP, TCEP, tris(2-butoxyethyl) phosphate (TBOEP) and 2-ethylhexyl diphenyl phosphate (EHDPP) were found, with DFs of 90%, 50%, 63.33% and 6.67%, respectively. TCPP, TCEP, triethyl phosphate (TEP) and triphenylphosphine oxide (TPPO) with DFs of 100%, 28.57%, 85.71% and 4.76% were found in plasma samples. The high DFs of TCPP and TCEP may be related to their high water solubility and hydrophilicity [16]. Other alkyl and aromatic OPEs were not detected in urine and plasma because the DFs were low, which is related to the rapid degradation and metabolism of alkyl and aromatic OPEs into phosphate diesters in the human body, making them difficult to detect [17]. Due to the low DFs, EHDPP and TPPO were not analyzed in this study. TCPP had the highest concentration ( geometric mean(gm) 1.07 ng/mL)followed by TCEP (gm 0.47 ng/mL) and TBOEP (gm 0.12 ng/mL) in urine samples. In plasma samples, TCPP had the highest concentration (gm 15.89 ng/mL) followed by TEP (gm 3.59 ng/mL) and TCEP (gm 0.39 ng/mL). Apparently, TCPP had the highest concentration in urine and plasma samples. Notably, a novel aryl-OPE, TPPO, is widely used as a synthetic intermediate in pharmaceutical products, and also as a ligand for various transitional metals. In recent years, it has begun to be widely detected in various environments [18,19], but no related previous reports have been seen in human urine, blood, plasma or serum. In this study, TPPO was first detected in one serum sample with a value of 12.68 ng/mL. This indicates that TPPO is beginning to be exposed in humans and that people are facing TPPO exposure. Considering the structural similarity of TPPO to triphenyl phosphate (TPHP), a proven endocrine disruptor [20], the health risk of TPPO warrants further investigation.

**Figure 1:**
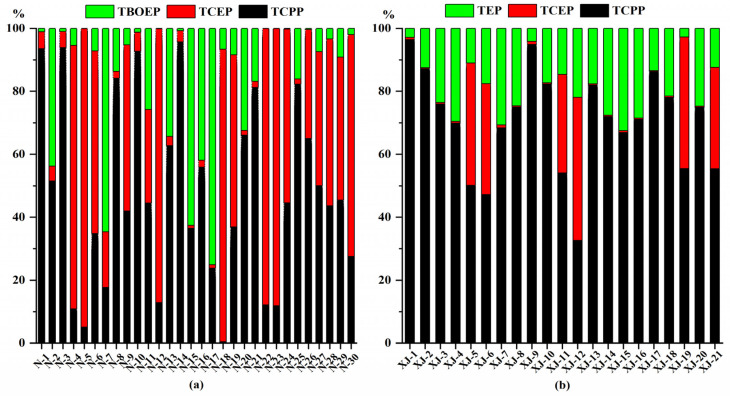
Composition of detectable OPEs in urine (**a**) and plasma samples (**b**).

The profiles of detectable OPEs in urine and plasma are shown in Figure 1. In urine samples, the average ratio of TCPP to the total concentration was 47.51%; for TCEP, it was 36.26%; and for TBOEP, it was 16.22%. In plasma samples, the average ratio of TCPP to the total concentration was 49.18%; for TEP, it was 12.63%; and for TCEP, it was 8.70%. It can be seen that chlorinated OPEs (TCPP, TCEP) are the main monomers of OPEs in both urine and plasma samples. TCEP and TCPP are widely used as substitutes for pentabromodiphenyl ether as flame retardants for flexible and rigid polyurethane foams, rubber and fabric coatings. Due to carcinogenicity, there has been a gradual restriction of TCEP in some developed countries, and TCEP has been gradually replaced by TCPP in recent years [21]. A previously study on OPEs in the urine of primary school students and pregnant women in Shenzhen revealed that the DFs, concentrations and profiles of TCEP were higher than those TCPP [13]. On the contrary, in this study, TCPP was higher than TCEP, which indicates that TCPP is widely used, and that there is widespread exposure to TCPP in industrial areas in Shenzhen.

### 3.2. MOPEs in Plasma and Recommendations for Biomarkers

Thus far, data on the concentration of OPE metabolites (mOPEs) in human blood/serum or plasma samples have remained limited [14]. In this study, we analyzed five mOPEs corresponding to their parent TnBP, TEP or TBOEP in plasma samples, and the results are shown in Table 2. As shown in Table 2, TBOEP and its three corresponding metabolites, BBOEP, BBOEHP and 3-OH-TBOEP, were not detectable. The metabolite–parent pairs with sufficient DFs (>50%) were TnBP–DnBP and TEP–DEP.

For TnBP–DnBP, TnBP was not detected in all plasma samples, but DnBP, in contrast, was widely detected, with a median of 9.92 ng/mL. Zhao et al. reported that TnBP (the parent compound of DnBP) had the highest level and was the main monomer in the blood/serum (median 37.8 ng/mL) from 257 participants recruited in Shenzhen [5]. Zhang et al. recently reported that urine DnBP (median 3.1 ng/mL) in Shenzhen had the highest level and was the main monomer detected in eight cities [22]. These findings indicate that Shenzhen is exposed to a high level of DnBP. DnBP is industrially produced and used for metal extraction, as a plasticizer and as an additive in the textile industry [23]. In recent studies, DnBP was found at higher concentrations than its parent compound (TnBP) in house dust in Guangzhou, at up to 163 ng/g. The high concentrations of DnBP in plasma samples in the present study may be a result of both DnBP metabolism in the body and direct exposure to DnBP via dust and dietary ingestion [24].

**Figure 2:**
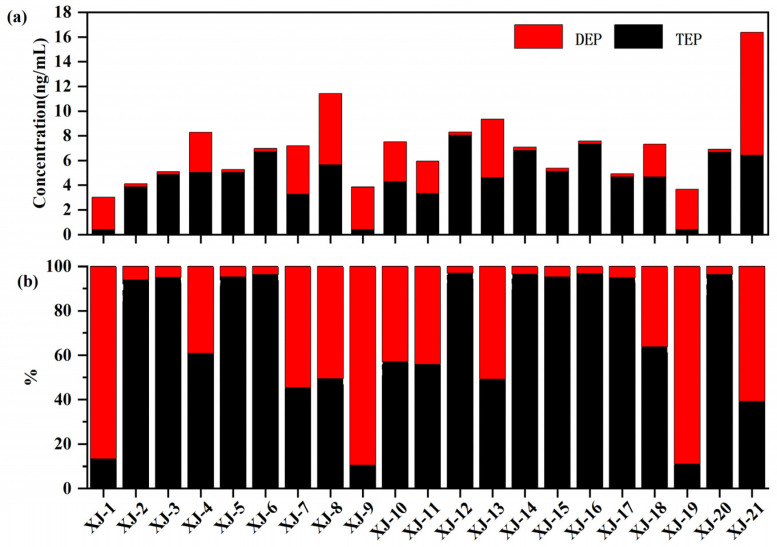
Concentration (**a**) and composition (**b**) of TEP and DEP in plasma samples.

For TEP–DEP, DEP (DF 52.38%, median 2.62 ng/mL) was found at lower concentrations than its parent compound (TEP) (DF 85.71%, median 4.85 ng/mL) (see Figure 2a). As can be seen in Figure 2b, the profiles of DEP in 7 of the 21 plasma samples were higher than for its parent compound (TEP), indicating that the DEP in plasma samples may come from direct ingestion from the environment. In recent studies, DEP was found in an effluent of an industrial sewage treatment plant [25] and the water of Dongting Lake, China [26]. Above all, DnBP and DEP in plasma samples may come from other sources in addition to OPE metabolism in the body and can be used as alternative exposure biomarkers in future.

### 3.3. Global and Regional Comparison of OPEs and Health Risk Assessment

The concentrations of OPEs in urine and plasma samples measured in our study were compared to previous studies in several locations (Table 3). In urine samples, TCPP (geometric mean (gm) 1.07 ng/mL) and TCEP (gm 0.47 ng/mL) were significantly higher than those reported in Australia and Beijing, Hong Kong and Jinan in China (Table 3). TBOEP (gm 0.12 ng/mL) was lower than in Australia (gm 0.59 ng/mL), but significantly higher than in Beijing, Hong Kong and Jinan in China. In plasma samples, TCPP (median 17.21 ng/mL) in this study was significantly lower than in Spain (median 93.9 ng/mL), but higher than in other reported regions (Table 3). TEP (median 4.85 ng/mL) had the highest value, 10 times higher than that of other studies. Above all, the exposure levels of TCEP, TCPP and TEP to the residents from industrial areas manufacturing large amounts of OPEs or OPE-related products in Shenzhen may be higher than those of other regions. TCPP, TCEP and TEP have been proved to have potential toxic and health effects in a number of studies [3,27], and the high level of exposure in this area deserves attention.

**Figure 3:**
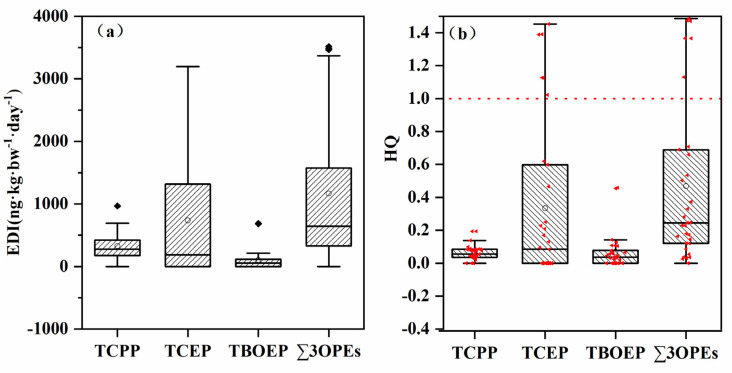
The estimated daily intake (EDI) (**a**) and potential health risk (HQ) values (**b**) of detectable OPEs in urine in this study.

The estimated daily intake (EDI) and potential health risk (HQ) values were calculated based on TCPP, TCEP and TBOEP in urine, and the results are shown in Figure 3. The HQ value (Figure 3b) shows that the HQ of TCPP and TBOEP is 0–0.19 and 0–0.45, respectively, both far below 1. The highest HQ value was found for TCEP, with a 95% percentile value of 1.23, higher than 1, revealing a moderate to high potential health risk from TCEP exposure. Compared with other OPEs, TCEP is more persistent. Due to potential toxicity and health effects, restrictions or prohibitions of TCEP have been issued in Canada, the United States and some European countries [1]. However, as a high-yield chlorinated OPE, TCEP is still widely used in some industrialized areas in China, and its health effects call for more attention.

## 4. Conclusions

In summary, this study reported on the OPE internal exposure and associated health risk of residents from a typical industrialized area in Shenzhen, China. TCPP and TCEP are the predominant OPEs and were found at concentrations higher than or comparable to those in other studies. In addition, DnBP and DEP were frequently detected in plasma samples and could be considered as biomarkers. Notably, the 95th HQ value of TCEP was higher than 1, revealing a potential health risk from TCEP exposure. In conclusion, human exposure to chlorinated OPEs (TCPP, TCEP) in industrialized areas calls for further investigation. The main limitation of this study lies in the small sample size. Further studies are needed to better assess OPE exposure in industrial areas with larger populations.

## Figures and Tables

**Table 1:** Summary of detectable OPE concentrations in urine and plasma samples (ng/mL).

	DF(%)	Median	Mean	Geometric Mean	Standard Deviation	Min	Max
**Urine Samples (*n* = 30)**							
TCPP	90	1.35	1.52	1.07	0.99	n.d.	4.79
TCEP	50	n.d.	3.37	0.47	5.19	n.d.	15.82
TBOEP	63.33	0.26	0.51	0.12	0.84	n.d.	3.41
EHDPP	6.67	n.d.	0.60	n.d.	0.1	n.d.	0.67
**Plasma Samples (*n* = 21)**							
TCPP	100	17.21	17.09	15.89	6.49	7.27	29.92
TCEP	28.57	n.d.	3.79	n.d.	6.5	n.d.	17.77
TEP	85.71	4.85	4.64	3.59	2.18	n.d.	8.04
TPPO	4.76	n.d.	n.d.	n.d.	-	n.d.	12.68

DF: detection frequency. n.d.: <MQL.

**Table 2:** mOPEs and their parent OPEs in plasma samples considered in the present study.

Parent OPEs	DF (%)	Mean (Min–Max)	mOPEs	Properties	DF (%)	Mean (Min–Max)
TnBP	0	n.d.	Di-n-butyl phosphate(DnBP)	Dialkyl metabolites	85.71	8.22 (n.d.–13.78)
TEP	85.71	4.64 (n.d.–8.04)	Diethyl phosphate(DEP)		52.38	2.29 (n.d.–9.96)
TBOEP	0	n.d.	Bis(2-butoxyethyl) phosphate (BBOEP)	Dialkyl metabolites	0	n.d.
			Bis(2-butoxyethyl)2-hydroxyethyl phosphate trimester (BBOEHP)	Hydroxylated metabolites	0	n.d.
			Bis(2-butoxyethyl)2-(3-hydroxybutoxy)ethyl phosphate trimester (3-OH-TBOEP)		0	n.d.

DF: detection frequency. n.d.: <MQL.

**Table 3:** Comparison of detected OPEs in urine and plasma in this study with other studies.

Region	Time	TCEP	TCIPP	TDCPP	TPHP	TEP	TBOEP	EHDPP	Reference
**Urine (Geometric Mean, ng/mL)**									
Shenzhen, China	2020	0.47	1.07	n.d	n.d	n.d	0.12	n.d	This study
Beijing, China	2018	n.d	n.d	n.d	n.d	0.075	0.038	n.d	[14]
Hongkong, China	2016	0.015	0.021	0.06	0.46	-	-	0.54	[28]
Jinan, China	2018	0.298	0.743	-	0.4	-	-	0.209	[24]
Australia	2014	n.d	n.d	n.d	n.d	n.d	n.d	n.d	[29]
Australia	2015	n.d	n.d	0.024	-	-	0.59	n.d	[30]
**Blood/Serum or Plasma (Median, ng/mL)**									
Shenzhen, China	2020	n.d	17.21	n.d	n.d	4.85	n.d	n.d	This study
Shenzhen, China	2012	n.d	0.71	n.d.	0.43	0.49	0.54	1.22	[5]
Hebei, China	2017	0.18	0.25	n.d	0.46	-	n.d.	0.78	[31]
Jinan, China	2018	0.3	0.74	0.11	0.4	0.14	-	0.21	[24]
Beijing, China	2018	n.d	n.d	n.d	0.37	0.43	0.16	1.1	[14]
Jiangsu, China	2013	0.1	0.05	n.d	0.35	0.15	0.05	0.85	[32]
Shandong, China	2018	214	n.d.	-	-	n.d.	-	7.2	[33]
Spain	2016	3.69	93.9	n.d.	22.7	n.d.	56.4	425.8	[34]

-: no analysis, n.d.: <MQL.

## Data Availability

Not applicable.

## Appendix A

**Table A1:** Detailed information of the study population (*n* = 51).

Category		Number (Percentage)
Age	<18	6 (11.76%)
	18–44	37 (72.55%)
	45–59	7 (13.73%)
	>59	1 (1.96%)
Gender	Male	12 (23.53%)
	Female	39 (76.47%)

**Table A2:** Abbreviations, CAS numbers, molecular formulas, molar masses and manufacturers of targeted OPEs and di-OPE compounds in this study.

No.	Compound	Abbreviation	Molecular Formula	CAS No.	Manufacturers
1	Tris(2-butoxyethyl) phosphate	TBOEP	C_18_H_39_O_7_P	78-51-3	Dr. Ehrenstorfer GmbH (Augsburg, Germany)
2	Trimethyl phosphate	TMP	C_3_H_9_O_4_P	512-56-1	AccuStandard (New Haven, CT, USA)
3	Tri-n-butyl phosphate	TnBP	C_12_H_27_O_4_P	126-73-8	Dr. Ehrenstorfer GmbH (Augsburg, Germany)
4	2-Ethylhexyl diphenyl phosphate	EHDPP	C_20_H_27_O_4_P	1241-94-7	
5	Tri-iso-butyl phosphate	TIBP	C_12_H_27_O_4_P	126-71-6	ANPEL Laboratory Technologies Inc. (Shanghai, China)
6	Triethyl phosphate	TEP	C_6_H_15_O_4_P	78-40-0	AccuStandard (New Haven, CT, USA)
7	Tetraphenyl m-phenylene bis(phosphate)	RDP	C_30_H_20_O_8_P2	57583-54-7	
8	Tripentyl phosphate	TNP	C_15_H_33_O_4_P	2528-38-3	
9	Tripropyl phosphate	TPrP	C_9_H_21_O_4_P	513-08-6	Dr. Ehrenstorfer GmbH (Augsburg, Germany)
10	Triphenyl phosphate	TPHP	C_18_H_15_O_4_P	115-86-6	
11	Triphenylphosphine Oxide	TPPO	C_18_H_15_OP	791-28-6	Toronto Research Chemicals Inc. (Toronto, ON, Canada)
12	Cresyl Diphenyl Phosphate	CDP	C_19_H_17_O_4_P	26444-49-5	Dr. Ehrenstorfer GmbH (Augsburg, Germany)
13	Tris(2-ethylhexyl) phosphate	TEHP	C_24_H_51_O_4_P	78-42-2	
14	Tris(2-chloropropyl) phosphate	TCPP	C_9_H_18_CL_3_PO_4_	13674-84-5	AccuStandard (New Heavn, CT, USA)
15	Tris(1,3-dichloro-2-propyl) phosphate	TDCIPP	C_9_H_15_C_l6_O_4_P	13674-87-8	
16	Tris(2-chloroethyl) phosphate	TCEP	C_6_H_12_C_l3_O_4_P	115-96-8	Dr. Ehrenstorfer GmbH (Augsburg, Germany)
17	Triisopropyl phosphate	TiPrP	C_9_H_21_O_4_P	513-02-0	AccuStandard (New Heavn, CT, USA)
18	Diethyl phosphate	DEP	C_4_H_11_O_4_P	598-02-7	
19	Di-n-butyl phosphate	DnBP	C_8_H_19_O_4_P	107-66-4	Dr. Ehrenstorfer GmbH (Augsburg, Germany)
20	Bis(2-butoxyethyl)2-hydroxyethyl phosphate triester	BBOEHP	C_14_H_31_O_7_P	1477494-86-2	Toronto Research Chemicals Inc. (Toronto, ON, Canada)
21	Bis(butoxyethyl)phosphate	BBOEP	C_12_H_27_O_6_P	14260-97-0	
22	Bis(2-butoxyethyl)2-(3-hydroxybutoxy)ethyl phosphate ttriester	3-OH-TBOEP	C_18_H_39_O_8_P	1477494-87-3	

**Table A3:** Liquid chromatography solvent gradient elution procedure.

Time (min)	Mobile Phase A (%)	Mobile Phase B (%)
0	30	70
2	60	40
22	85	15
24	98	2
25	98	2
30	30	70

**Table A4:** Detailed information of the mass spectrum parameters.

Analyte	Precursor Ion (*m*/*z*)	Fragment (V)	Product Ion (*m*/*z*)	CE (V)
TBOEP	399	124	45/57	25/37
TMP	141	96	109/47	17/29
TnBP	267	81	99/211	13/5
EHDPP	251	117	51/77	69/33
TIBP	267	81	99/81	13/61
TEP	183	61	99/81	17/49
RDP	575	210	77/152	85/70
TNP	309	101	99/81	17/77
TPrP	225	76	99/141	13/5
TPHP	327	157	77/152	49/45
TPPO	279	170	201/77	25/53
CDP	341	175	91/65	37/73
TEHP	434	130	99/113	40/10
TCPP	327	100	99/175	18/10
TDCIPP	431	110	99/209	15/12
TCEP	285	85	99/63	20/26
TiPrP	225	64	99/81	13/57
DEP	155	75	99/127	13/5
DnBP	211	75	99/63	9/89
BBOEHP	343	95	45/243	17/9
BBOEP	299	95	45/199	17/9
3-OH-TBOEP	415	105	45/55	25/37
TBOEP-d_27_	426	105	46/66	25/37
TMP-C_13_	144	90	111/80	20/40
TnBP-d_27_	294	95	102/83	17/77

**Table A5:** Recoveries (%), method detection limits (MDLs, g/mL) and method quantitation limits (MQLs, ng/mL) of the target analytes in urine and plasma samples.

Analyte	Recoveries		Urine		Plasma	
	Urine	Plasma	MDLs	MQLs	MDLs	MQLs
TBOEP	85.4 ± 20.3	86.9 ± 14.4	0.05	0.2	0.2	0.8
TMP	82.3 ± 17.4	84.3 ± 12.1	0.05	0.2	0.2	0.8
TnBP	76.5 ± 10.4	86.1 ± 15.3	0.05	0.2	0.2	0.8
EHDPP	91.0 ± 22.6	79.6 ± 13.4	0.1	0.4	0.4	1.5
TIBP	84.8 ± 17.4	84.5 ± 16.7	0.05	0.2	0.2	0.8
TEP	93.1 ± 20.2	83.1 ± 18.3	0.1	0.4	0.4	1.5
RDP	85.6 ± 18.8	89.2 ± 22.6	0.2	0.8	0.8	3
TNP	79.5 ± 12.9	76.8 ± 12.6	0.1	0.4	0.4	1.5
TPrP	86.7 ± 21.3	87.9 ± 23.7	0.02	0.08	0.1	0.4
TPHP	89.6 ± 20.8	89.7 ± 19.5	0.02	0.08	0.1	0.4
TPPO	68.3 ± 10.1	86.1 ± 15.3	0.05	0.2	0.2	0.8
CDP	78.8 ± 15.5	90.3 ± 23.2	0.2	0.8	0.8	3
TEHP	73.8 ± 16.2	86.8 ± 16.2	0.1	0.4	0.4	1.5
TCPP	76.2 ± 8.6	91.8 ± 21.3	0.2	0.8	0.8	3
TDCIPP	81.1 ± 15.2	103.2 ± 16.8	0.3	1	1	4
TCEP	103.1 ± 23.2	107.3 ± 24.0	0.3	1	1	4
TiPrP	79.2 ± 16.6	84.9 ± 14.3	0.02	0.08	0.08	0.4
DEP	89.3 ± 13.3	89.1 ± 8.3	0.08	0.3	0.3	1
DnBP	76.3 ± 5.6	79.3 ± 5.4	0.05	0.2	0.2	0.8
BBOEHP	87.2 ± 7.9	76.8.4 ± 3.6	0.05	0.2	0.2	0.8
BBOEP	78.4 ± 14.3	82.6 ± 11.3	0.05	0.2	0.2	0.8
3-OH-TBOEP	84.5 ± 15.2	82.9 ± 12.6	0.04	0.15	0.15	0.6

## References

[1] Chokwe T.B., Abafe O.A., Mbelu S.P., Okonkwo J.O., Sibali L.L. (2020). A review of sources, fate, levels, toxicity, exposure and transformations of organophosphorus flame-retardants and plasticizers in the environment. Emerg. Contam..

[2] He J., Wang Z., Zhao L., Ma H., Huang J., Li H., Mao X., Huang T., Gao H., Ma J. (2021). Gridded emission inventory of organophosphorus flame retardants in China and inventory validation. Environ. Pollut..

[3] Du J., Li H., Xu S., Zhou Q., Jin M., Tang J. (2019). A review of organophosphorus flame retardants (OPFRs): Occurrence, bioaccumulation, toxicity, and organism exposure. Environ. Sci. Pollut. Res..

[4] Hu Y.-X., Sun Y.-X., Li X., Xu W.-H., Zhang Y., Luo X.-J., Dai S.-H., Xu X.-R., Mai B.-X. (2017). Organophosphorus flame retardants in mangrove sediments from the Pearl River Estuary, South China. Chemosphere.

[5] Qin W.Y., Mao L.S., Chen Y.H., Liu G.H., Luo X.U., Jang J., Guan Y.F. (2019). Distribution characteristics and health risk assessment of five organophosphorus flame retardants in indoor dust in Shenzhen. J. Environ. Health.

[6] Chen Y., Fang J., Ren L., Fan R., Zhang J., Liu G., Zhou L., Chen D., Yu Y., Lu S. (2018). Urinary metabolites of organophosphate esters in children in South China: Concentrations, profiles and estimated daily intake. Environ. Pollut..

[7] Chen Y., Jiang L., Lu S., Kang L., Luo X., Liu G., Cui X., Yu Y. (2019). Organophosphate ester and phthalate ester metabolites in urine from primiparas in Shenzhen, China: Implications for health risks. Environ. Pollut..

[8] Zhao F., Wan Y., Zhao H., Hu W., Mu D., Webster T.F., Hu J. (2016). Levels of Blood Organophosphorus Flame Retardants and Association with Changes in Human Sphingolipid Homeostasis. Environ. Sci. Technol..

[9] Luo K., Zhang R., Aimuzi R., Wang Y., Nian M., Zhang J. (2020). Exposure to Organophosphate esters and metabolic syndrome in adults. Environ. Int..

[10] Ingle M.E., Watkins D., Rosario Z., Vélezvega C.M., Calafat A.M., Ospina M., Ferguson K., Cordero J.F., Alshawabkeh A., Meeker J.D. (2020). An exploratory analysis of urinary organophosphate ester metabolites and oxidative stress among pregnant women in Puerto Rico. Sci. Total Environ..

[11] Yao Y., Li M., Pan L., Duan Y., Duan X., Li Y., Sun H. (2021). Exposure to organophosphate ester flame retardants and plasticizers during pregnancy: Thyroid endocrine disruption and mediation role of oxidative stress. Environ. Int..

[12] Liu W., Luo D., Xia W., Tao Y., Wang L., Yu M., Hu L., Zhou A., Covaci A., Lin C. (2021). Prenatal exposure to halogenated, aryl, and alkyl organophosphate esters and child neurodevelopment at two years of age. J. Hazard. Mater..

[13] Liu Y., Li Y., Dong S., Han L., Guo R., Fu Y., Zhang S., Chen J. (2021). The risk and impact of organophosphate esters on the development of female-specific cancers: Comparative analysis of patients with benign and malignant tumors. J. Hazard. Mater..

[14] Hou M., Shi Y., Jin Q., Cai Y. (2020). Organophosphate esters and their metabolites in paired human whole blood, serum, and urine as biomarkers of exposure. Environ. Int..

[15] Zhang T., Bai X.-Y., Lu S.-Y., Zhang B., Xie L., Zheng H.-C., Jiang Y.-C., Zhou M.-Z., Zhou Z.-Q., Song S.-M. (2018). Urinary metabolites of organophosphate flame retardants in China: Health risk from tris(2-chloroethyl) phosphate (TCEP) exposure. Environ. Int..

[16] Van der Veen I., de Boer J. (2012). Phosphorus flame retardants: Properties, production, environmental occurrence, toxicity and analysis. Chemosphere.

[17] Wang Y., Li W., Martinez-Moral M.-P., Sun H., Kannan K. (2019). Metabolites of organophosphate esters in urine from the United States: Concentrations, temporal variability, and exposure assessment. Environ. Int..

[18] Chen M.-H., Ma W.-L. (2021). A review on the occurrence of organophosphate flame retardants in the aquatic environment in China and implications for risk assessment. Sci. Total Environ..

[19] Li J., Li W., Gao X., Liu L., Shen M., Chen H., Zhu M., Zeng L., Zeng E.Y. (2020). Occurrence of multiple classes of emerging photoinitiators in indoor dust from E-waste recycling facilities and adjacent communities in South China and implications for human exposure. Environ. Int..

[20] Luo D., Liu W., Wu W., Tao Y., Hu L., Wang L., Yu M., Zhou A., Covaci A., Xia W. (2021). Trimester-specific effects of maternal exposure to organophosphate flame retardants on offspring size at birth: A prospective cohort study in China. J. Hazard. Mater..

[21] Iqbal M., Syed J.H., Katsoyiannis A., Malik R.N., Farooqi A., Butt A., Li J., Zhang G., Cincinelli A., Jones K.C. (2017). Legacy and emerging flame retardants (FRs) in the freshwater ecosystem: A review. Environ. Res..

[22] Zhang X.H., Zhao F.R., Hu J.Y. (2021). Exposure assessment and health risk of organophosphate flame retardants in general population in China. Asian J. Ecotoxicol..

[23] Quintana J.B., Rodil R., Reemtsma T., García-López M., Rodríguez I. (2008). Organophosphorus flame retardants and plasticizers in water and air II. Analytical methodology. TrAC Trends Anal. Chem..

[24] Hou M., Fang J., Shi Y., Tang S., Dong H., Liu Y., Deng F., Giesy J.P., Pollitt K.J.G., Cai Y. (2021). Exposure to organophosphate esters in elderly people: Relationships of OPE body burdens with indoor air and dust concentrations and food consumption. Environ. Int..

[25] Xu L., Hu Q., Liu J., Liu S., Liu C., Deng Q., Zeng X., Yu Z. (2019). Occurrence of organophosphate esters and their diesters degradation products in industrial wastewater treatment plants in China: Implication for the usage and potential degradation during production processing. Environ. Pollut..

[26] Xu L., Zhang B., Hu Q., Liu Y., Shang T., Zeng X., Yu Z. (2021). Occurrence and spatio-seasonal distribution of organophosphate tri- and di-esters in surface water from Dongting Lake and their potential biological risk. Environ. Pollut..

[27] Wang C., Chen H., Li H., Yu J., Wang X., Liu Y. (2020). Review of emerging contaminant tris(1,3-dichloro-2-propyl)phosphate: Environmental occurrence, exposure, and risks to organisms and human health. Environ. Int..

[28] Li N., Ho W., Wu R.S.S., Ying G.-G., Wang Z., Jones K.C., Deng W.-J. (2019). Organophosphate flame retardants and bisphenol A in children’s urine in Hong Kong: Has the burden been underestimated?. Ecotoxicol. Environ. Saf..

[29] He C., English K., Baduel C., Thai P., Jagals P., Ware R., Li Y., Wang X., Sly P., Mueller J. (2018). Concentrations of organophosphate flame retardants and plasticizers in urine from young children in Queensland, Australia and associations with environmental and behavioural factors. Environ. Res..

[30] He C., Toms L.-M., Thai P., Eede N.V.D., Wang X., Li Y., Baduel C., Harden F.A., Heffernan A., Hobson P. (2018). Urinary metabolites of organophosphate esters: Concentrations and age trends in Australian children. Environ. Int..

[31] Wang X., Shan G., Zhu L. (2020). Estimation of internal human daily intakes of organophosphate esters using one-compartment toxicokinetic model in the whole blood from Hebei Province, China. Environ. Res..

[32] Ya M., Yu N., Zhang Y., Su H., Tang S., Su G. (2019). Biomonitoring of organophosphate triesters and diesters in human blood in Jiangsu Province, eastern China: Occurrences, associations, and suspect screening of novel metabolites. Environ. Int..

[33] Gao D., Yang J., Bekele T.G., Zhao S., Zhao H., Li J., Wang M., Zhao H. (2020). Organophosphate esters in human serum in Bohai Bay, North China. Environ. Sci. Pollut. Res..

[34] Henríquez-Hernández L.A., Carretón E., Camacho M., Montoya-Alonso J.A., Boada L.D., Martín V.B., Cordón Y.F., Cordón S.F., Zumbado M., Luzardo O.P. (2017). Potential role of pet cats as a sentinel species for human exposure to flame retardants. Front. Vet. Sci..

